# The feasibility of meeting the WHO guidelines for sodium and potassium: a cross-national comparison study

**DOI:** 10.1136/bmjopen-2014-006625

**Published:** 2015-03-20

**Authors:** Adam Drewnowski, Colin D Rehm, Matthieu Maillot, Alfonso Mendoza, Pablo Monsivais

**Affiliations:** 1 Center for Public Health Nutrition, University of Washington, Seattle, Washington, USA; 2 Institute for Cardiometabolism and Nutrition (ICAN), Université Pierre et Marie Curie Paris VI, Groupe Hospitalier Pitié-Salpetrière, Paris, France; 3 Friedman School of Nutrition Science and Policy, Tufts University, Boston, Massachusetts, USA; 4 MS-Nutrition SAS, Marseille, France; 5 Departamento de Ciencias Sociales, Centro de Investigación e Inteligencia Económica, Universidad Popular Autónoma del Estado de Puebla, Puebla, Mexico; 6 The Centre for Diet and Activity Research (CEDAR), University of Cambridge, Cambridge, UK

**Keywords:** EPIDEMIOLOGY, PUBLIC HEALTH

## Abstract

**Objective:**

To determine joint compliance with the WHO sodium–potassium goals in four different countries, using data from nationally representative dietary surveys.

**Setting:**

Compared to national and international recommendations and guidelines, the world's population consumes too much sodium and inadequate amounts of potassium. The WHO recommends consuming less than 2000 mg sodium (86 mmol) and at least 3510 mg potassium (90 mmol) per person per day.

**Participants:**

Dietary surveillance data were obtained from the National Health and Nutrition Examination Survey (NHANES 2007–2010) for the USA; the Encuesta Nacional de Salud y Nutrición 2012 for Mexico; the Individual and National Study on Food Consumption (INCA2) for France; and the National Diet and Nutrition Survey (NDNS) for the UK.

**Primary outcome measures:**

We estimated the proportion of adults meeting the joint WHO sodium–potassium goals in the USA, the UK, France and Mexico.

**Results:**

The upper bounds of joint compliance with the WHO sodium–potassium goals were estimated at 0.3% in the USA, 0.15% in Mexico, 0.5% in France and 0.1% in the UK.

**Conclusions:**

Given prevailing food consumption patterns and the current food supply, implementing WHO guidelines will be an enormous challenge for global public health.

**Strengths and limitations of this study**

- The present data represent the best source of information on the dietary habits of a large and nationally representative sample of adults in each country.
- Each of the data sets analysed is a flagship national study that informs a nation's food and nutrition policy and provides data to be furnished to the WHO.
- Different dietary surveys used different methodologies and nutrient composition databases.
- Analyses were based on a limited number of days, which may not capture habitual intakes.
- All nutrient intakes were based on self-reports and were subject to reporting errors that may result in under-estimating the consumption of sodium. In addition, sodium intakes from table salt and supplements were not included.

## Introduction

Compared to international and national recommendations and guidelines, the world's population consumes too much dietary sodium and inadequate amounts of potassium.^1–4^ Excessive sodium consumption and excessively high dietary sodium–potassium ratios have been linked to a higher risk of cardiovascular disease (CVD), including coronary heart disease and stroke.^5–7^ Annually, 1.65 million deaths from cardiovascular disease, or about 10% of cardiovascular deaths, could be attributed to excess sodium intake, globally.^8^

Preventing non-communicable diseases is a top priority for global public health.^1–3^ The WHO has recommended consuming less than 2000 mg of sodium, or 5 g of salt, and consuming at least 3510 mg of potassium per day.^3^ These amounts are equivalent to 86 mmol sodium and 90 mmol potassium, making for a desirable dietary sodium–potassium ratio of approximately 1.0.^9^

Current consumption levels are vastly different.^9^ ^10^ Median sodium intakes in the USA among adults were estimated at 3371 mg/day, while potassium intakes were estimated at 2631 mg/day.^11^ Relative to recommended values of 2300 and 1500 mg/day for sodium, 90.7% and 99.4% consumed excess sodium, respectively, while only 1.4% consumed more than 4700 mg/day of potassium. Sodium intakes in the UK and France were >50% above the WHO recommended values.^12^ ^13^ Assessments of urinary-based measures of sodium paint a similarly dire picture; estimates show that global intakes are approximately 3950 mg/day, nearly twice the recommended value.^4^ In no region did average sodium intakes fall below 2000 mg/day.^4^

Reducing sodium while simultaneously increasing potassium intakes presents multiple challenges.^14^ ^15^ First, sodium and potassium intakes are closely tied to energy balance, such that persons with higher energy requirements consume more potassium but also more sodium.^16^ Very low sodium diets tend to be low in calories and may not provide adequate nutrition.^15^ Uncoupling sodium from dietary potassium can be problematic, since both are present in many of the same foods.^17^ In mathematical models based on dietary intakes from the National Health and Nutrition Examination Survey (NHANES), the US sodium and potassium guidelines appeared to be incompatible with each other.^14^ In empirical analyses of the 2003–2008 NHANES data, joint compliance with the published US sodium–potassium guidelines (1500 mg sodium and 4700 mg potassium) was estimated between 0 and 0.015%.^16^

We now examined joint compliance with the new WHO sodium and potassium guidelines in four different countries, using nationally representative databases. Since the WHO guidelines^1–3^ have an intended global reach, examining sodium and potassium intakes in more than one country is of particular interest. Assessing the feasibility of dietary recommendations and guidelines is a critical component of global public health policy.

## Methods

Data on sodium and potassium intakes were obtained from publicly available national dietary surveys for the US, Mexico, France and the UK. Each of those studies was approved by institutional review boards in their respective countries. Detailed procedures for data collection have been published previously and are noted below.

The National Health and Nutrition Examination Survey (NHANES 2007–2010) is a nationally representative survey of the US population.^18^ The present analyses were based on 9720 adults aged ≥20 years with two complete and valid 24 h recalls. For each survey participant, sodium and potassium intakes were obtained from the mean of two non-consecutive 24 h recalls. Although two dietary recalls do not reflect true estimates of habitual intake, they do provide an upper bound of the proportion of the population meeting sodium and potassium goals.

The Encuesta Nacional de Salud y Nutrición 2012 (ENSANUT 2012) in Mexico is based on a random representative sample of children and adults.^19^ Dietary intake data are based on a semiquantitative and previously validated 7-day food frequency questionnaire.^20^ The present analyses were based on adults aged ≥20 only (n=2879). The databases and documentation of protocols are available online.^19^

The Individual and National Study on Food Consumption (INCA2) in France is based on a nationally representative sample of 1455 children ages 3–17 years and 2624 adults aged 18–79 years. The study was conducted by the ANSES government agency (Agence nationale de sécurité sanitaire de l’alimentation, de l’environnement et du travail).^12^ Dietary intake data were based on 7-day food diaries, aided by a photographic atlas of portion sizes. The present analyses were based on adults aged ≥20 years only, excluding under and over reporters (n=1726).

The National Diet and Nutrition Survey (NDNS) in the UK is a nationally representative sample of UK residents aged 1.5 years and older, stratified by age and gender. The survey recruits 1000 participants per year, children as well as adults.^21^ Data analysed in this study were for years 1–3 of the rolling programme, with 4-day food diaries available for 3073 individuals. The present analyses were based on a sample of 1472 adults. Further details on sampling, data collection and processing are published.^21^

The proportion of persons meeting the sodium and potassium guidelines, separately and jointly, was calculated.^16^ Survey weighted medians and 25th and 75th centiles for sodium and potassium intakes were calculated for each survey sample. All analyses of NHANES data were conducted using Stata V.13.0 (College Station, Texas, USA), accounting for the complex survey design.

## Results

**Figure 1:**
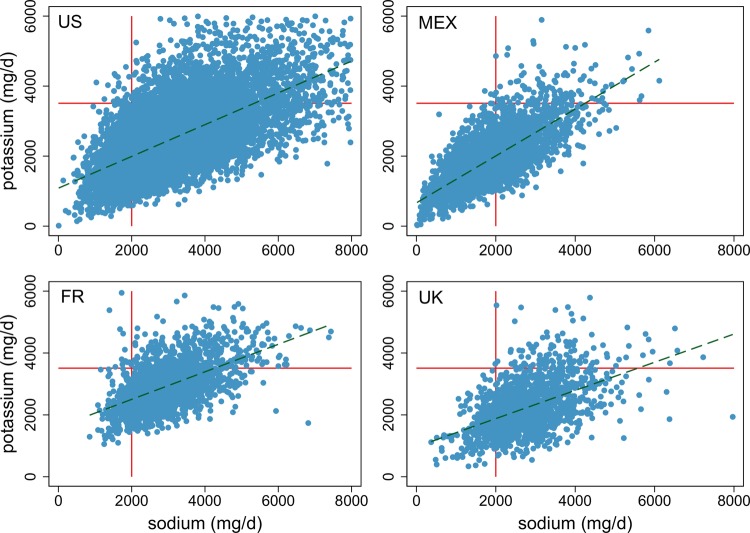
Distribution of sodium (x axis) and potassium intakes (y axis) in four countries relative to WHO sodium–potassium goals. The proportion of the population meeting the joint goals is shown in the top left hand quadrant of each panel.

Figure 1 shows the distributions of individual intakes of sodium and potassium as scatterplots, separately for each country. The top left panel shows mean 2-day sodium and potassium intakes for US adults aged ≥20 years in 2007–2010 NHANES. The figure was cropped to exclude potassium intakes of >6000 mg/day (n=87 adults) and sodium intakes of >8000 mg/day (n=99 adults). As indicated by solid lines, 86.2% of the sample failed to meet the WHO sodium goal (95% CI 84.9 to 87.4) and 80.4% failed to meet the WHO potassium goal (95% CI 79.1 to 81.7). The upper bound of joint compliance with the WHO sodium–potassium goals was estimated at 0.3% (95% CI 0.2 to 0.5).

Figure 1 (top right panel) shows estimated sodium and potassium intakes for 2879 Mexican adults aged ≥20 years in the 2012 ENSANUT. As indicated, 44% failed to meet the WHO sodium goal (95% CI 41% to 47.2%) and 95% failed to meet the WHO potassium goal (95% CI 93.4% to 96.2%). Joint compliance with WHO sodium–potassium goals was estimated at 0.15% (95% CI 0.04% to 0.5%).

Figure 1 (bottom left panel) shows estimated sodium and potassium intakes for 1726 French adults aged ≥20 years in the INCA 2 study. As indicated, 89.1% failed to meet the WHO sodium goal (95% CI 87.2 to 90.8%) and 77% failed to meet the potassium goal (95% CI 74.5 to 79.3%). Joint compliance with both WHO goals was estimated at 0.5% (95% CI 0.2 to 1.0).

Mean sodium and potassium intakes for 1472 UK adults aged ≥20 years in the NDNS study year are shown in figure 1 (bottom right panel). As indicated, 83.4% failed to meet the sodium goal (95% CI 81.3 to 85.5) and 91.9% failed to meet the potassium goal (95% CI 90.2 to 93.4). Joint compliance with both WHO goals was estimated at 0.1% (95% CI 0.02 to 0.9).

**Figure 2:**
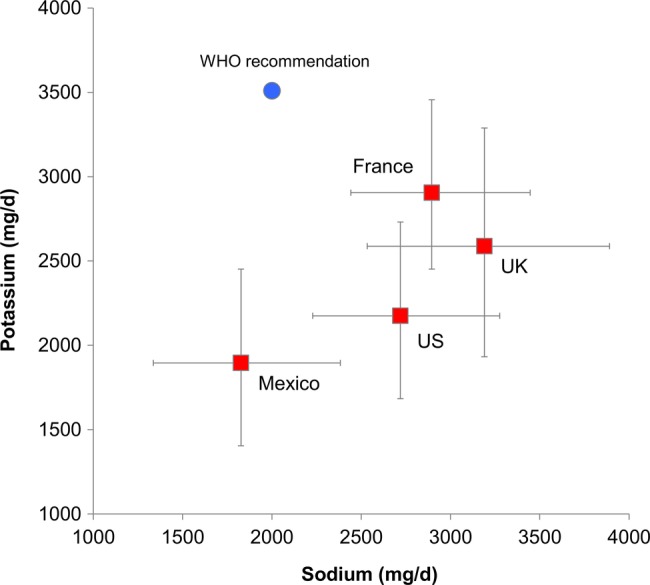
Relation between WHO recommendations and survey-weighted medians for sodium and potassium intakes for the four countries. Error bars correspond to IQR (25th and 75th centile).

Figure 2 shows the relation between the WHO sodium–potassium goals and survey-weighted medians and IQRs for each country.

## Discussion

This is the first analysis of the feasibility of the WHO joint goals, based on nationally representative dietary data from four countries. How the sodium and potassium goals are determined illustrates the complexities of translating science into public policy. The WHO global goals were based on the levels recommended by the 2002 joint WHO/FAO Expert Consultation and adopted by the WHO guidelines for CVD prevention.^22^

The 2010 US Dietary Guidelines set sodium targets at 2300 mg/day for the general population, with a lower goal of 1500 mg/day for certain subgroups at risk.^10^ The 1500 mg/day target was initially suggested by the Institute of Medicine (IOM) expert committee; however, issues were later raised about health benefits and likely compliance.^23^ Since then, the IOM has concluded that the evidence for the health benefits associated with sodium intakes <2300 mg/day was lacking.^23^ ^24^ Noting that very low sodium intakes may also increase health risks—particularly in certain groups—the IOM report reaffirmed the 2300 mg/day sodium goal for the general population.^23^ ^24^

That report was criticised by the American Heart Association (AHA) as being incomplete, and AHA continues to recommend consuming less than 1500 mg/day.^25^ ^26^ The 2010 Dietary Guidelines for Americans, which currently informs federal efforts regarding sodium intakes, recommends a sodium goal of 1500 mg/day for persons aged >50 years, African-Americans, and persons with diabetes, hypertension and chronic kidney disease.^10^ ^27^ Given the considerable discussion regarding these recommendations, the recommendations of the 2015 Dietary Guidelines for Americans Advisory Committee for sodium will be met with much anticipation. There has been much less debate regarding potassium goals, which remained at a relatively high 4700 mg/day, when compared to current levels of dietary potassium.^10^

The French Recommended Dietary Allowances (Apports Nutritionnels Conseillés, or ANC) set salt the consumption target in the 5–8 g/day range, equivalent to 2000–3200 mg/day of sodium. The potassium target was set at 3100 mg/day. The UK agency NICE has set UK sodium target at <2360 mg/day of sodium per adult by year 2015, to be reduced further to a very low <1180 mg/day of sodium by 2025.^9^ ^13^ ^28^ Potassium targets were not specified. The Pan American Health Organisation (PAHO) has been making signal efforts at sodium reduction in the Americas and is likely to follow the WHO lead on sodium and potassium goals, as is Mexico.

The present analyses, based on nationally representative studies, suggest that joint compliance with the published WHO sodium and potassium goals is close to zero. These conclusions are consistent with published analyses of the NHANES data by the Centers for Disease Control, which estimated that only 0.6% of the population met the 1500 mg/day sodium goal and 1.4% met the goal for potassium.^11^ The upper bounds of joint compliance with sodium–potassium, based on the same NHANES data, were estimated by us at 0.015% for 1500 mg/day sodium and at 0.12% for 2300 mg/day sodium.^16^

Given the surprisingly low levels of compliance with global goals, it is clear that purely educational and individual-level approaches to sodium reduction are unlikely to be successful. Reducing sodium in the food supply through thorough reformulation of processed and packaged foods, as well as by making parallel efforts to reduce the sodium content of foods prepared away from home, are the most promising strategies to reduce population-wide sodium exposure.^29^ ^30^ The largest contributors of sodium in the diet depend on food classifications evaluated, but yeast breads, pizza, processed meats, cheese and meat/poultry mixed dishes, are among the most important sources of dietary sodium in the USA.^31^ ^32^ Processed foods, including breads, cereals and grains, contribute a majority of sodium in the UK and in France as well.^12^ ^33^ It is important to note that frequency of consumption, rather than sodium density (eg, sodium per serving or per calorie), is the most important determinant of population-wide sodium reduction. Therefore, reformulation efforts, whether voluntary or mandated, should focus on the largest sources of sodium population-wide rather than the most sodium-rich products.

On the other hand, increasing potassium intakes cannot be achieved through reformulation. As in sodium, the most important sources of potassium are frequently consumed foods, including milk, coffee, mixed meat/chicken dishes, fruit juices and potatoes in the USA, and dairy products, vegetables and meats in France.^12^ ^34^ Achieving adequate potassium intakes will likely require increasing the consumption of less frequently consumed potassium-rich foods, including beans, dark-green vegetables, dried fruits and fish. While simultaneously decreasing sodium and increasing potassium should be the long-term goal for populations, independently increasing potassium intakes in diets will also likely have numerous benefits.^35–37^

Perceived or actual food costs may be one reason why the observed sodium–potassium ratio is excessively high.^38^ Merging dietary intakes from the 2001 to 2002 NHANES with a national food price database showed that more favourable sodium–potassium ratios were associated with higher per calorie diet costs.^17^ ^39^ The difference in diet cost among participants with highest and lowest potassium intakes was $1.49/day (95% CI 1.29 to 1.69), comparable to the $1.50/day amount associated with healthier diets from a recent meta-analysis of diet cost studies.^17^ ^40^ No association was observed between diet cost and sodium. These results suggest that, under current eating patterns, increasing potassium intakes may incur increasing dietary costs, while decreasing sodium intakes can likely be achieved in a cost neutral manner.

Calorie-indexed dietary guidelines could also help. Population-wide sodium and potassium goals continue to be framed in mg per person per day, regardless of individual energy needs.^14^ ^15^ Persons with high energy intakes may be able to meet potassium but not sodium goals; whereas persons with low energy intakes may meet sodium goals but have inadequate potassium, as sodium and potassium are both highly correlated with dietary energy. One way to address this issue may be to tie sodium and potassium goals to energy requirements. Such a nutrient indexing model was proposed in 1999 for the Dietary Approaches to Stopping Hypertension (DASH) diet^41^ and, more recently, by ANSES in France.^12^ Another approach would be to focus dietary guidance on the sodium–potassium ratio, currently much higher than the recommended target of 1.0.^6^

The present analysis of the multinational data sets has several limitations. First, the different dietary surveys used different methodologies, including dietary recalls (US), food diaries/records (UK and France) and a food frequency questionnaire (Mexico). Second, analyses were based on a limited number of days for those data derived from dietary recalls and food diaries, which may not capture habitual intakes. This is particularly a concern for the US data where a two-day average was used. Advanced methods are available to estimate habitual intakes based on dietary recalls for a single dietary constituent, though methods for estimating joint compliance based on habitual intakes have not been developed. As a result, our population estimates represent an upper bound of the compliance to sodium, potassium and the joint goals. Third, all nutrient intakes were based on self-reports and were subject to reporting errors that may result in under-estimating the consumption of sodium, due to omission of sodium-dense foods or potential over-reporting of potassium intakes, such as fruits/vegetables. Fourth, sodium intakes from table salt and supplements were not included. Lastly, the nutrient composition databases used by different countries may differ in quality. Despite these limitations, the present data represent the best source of information on the dietary habits of a large and nationally representative sample of adults in each country. Each of the data sets analysed is a flagship national study that informs that nation's food and nutrition policy and provides data to be furnished to the WHO.

There is a case to be made for more feasibility research in the formulation of public policy. First, the fact that the proposed WHO guidelines are very far removed from current patterns of intake in multiple countries should be an indication of potential problems ahead. Dealing with this level of non-compliance will be a challenge for global public health practice. Second, dietary guidelines need to suggest ways of meeting recommendations without substantially increasing diet costs. Third, given globalisation of science, expertise and access to data should be readily available. Yet different countries have different guidelines for sodium and potassium that do not necessarily correspond to those issued by the WHO. Better harmonisation of dietary goals would aid in the implementation of global food and nutrition policies.
